# Hæmatology—VI

**Published:** 1910-06-18

**Authors:** 


### PATHOLOGY.

### HÆMATOLOGY—VI.

### FIXING AND STAINING BLOOD.

For many reasons it may be necessary to examine the blood in a dried condition and suitably stained. This method does not, of course, replace the examination of fresh blood, it supplements it merely. Thin, well-spread-out films are required; these are usually now made on slides, the old cover-slip method having largely died out owing to the difficulty of staining and to the ease with which the covers got broken. Most people have their own special way of making films, and any of these, provided they produce good results, will do. One way is to put a drop of blood on a slide, lay a needle on this, and when the blood spreads out along it draw it (the needle) along the slide; another way is to catch a drop of blood on the edge of the end of a slide and then glide this along another slide. Once made and dried the slides can be stored in boxes or simply wrapped up in paper and kept till required; it must be remembered, however, that they will not keep indefinitely, and the sooner they are stained and prepared the better. (After six months they are of very little use.) The first procedure in the act of staining a film is to fix the blood on the slide, the reason of this necessity being that when a watery stain comes in contact with the red corpuscles the hæmoglobin is dissolved, and nothing would be left with the exception of the leucocytes. Many modes of fixing have from time to time been employed, namely, heat (roughly over a spirit-lamp, more accurately in a hot chamber at 120° C. for half an hour); absolute alcohol; absolute alcohol and ether, equal parts; osmic acid; bichloride of mercury, saturated solution; formalin, etc. A method recommended by Gulland combines the alcohol and ether with the mercury as follows:—

| | |
|---|---|
| Absolute alcohol ... ... ... | 25 c.c. |
| Pure ether ... ... ... ... | 25 c.c. |
| $HgCl_2$ (alcoholic solution) (2 grs. in 10 c.c. alcohol) ... ... ... | 5 drops. |

Films to be placed in this while wet for 5 minutes.

The best of all these is the absolute alcohol, or this with equal parts of ether, while in Leishman's stain the fixative is the methyl alcohol in which the stain is dissolved. Drop the slides into the alcohol and ether, leave for five minutes, take out and dry in the air; they are then ready for staining. Staining may be divided into (1) staining with simple stains; (2) staining with any of the modifications of Romanowsky's chromatin stain.

(1) Of simple stains it will be sufficient to mention borax methylene blue, carbol thionin, hæmatoxylin, hæmatoxylin and eosin, and hæmalam. Ehrlich's triple stain is practically never used now, so it will not be described. The formulæ for these stains are as follows:—

1. Borax Methylene Blue.

| | |
|---|---|
| Methylene blue ... ... ... ... | 2 grams. |
| Borax ... ... ... ... ... | 5 grams. |
| Aq. destillata ... ... ... ... | 100 c.c. |

Stain for 40 seconds, wash well in water. Red corpuscles should be greenish, the nuclei of the leucocytes a brilliant purple.

2. Carbol Thionin.

(a) Thionin blue ... ... ... ... 1 gram

Dissolve in 100 c.c. carbolic acid sol. 1-40. Dilute one volume with 3 of water and filter.

(b) *Another method.*

| | |
|---|---|
| Thionin crystals ... ... ... | 1.5 gram. |
| Absolute alcohol ... ... ... | 10 c.c. |
| Carbolic acid, 5 per cent. ... ... | 100 c.c. |

Dilute 1 in 3 for blood-staining.

Stain for 10 to 15 minutes; wash well in water; dry.

3. Hæmatoxylin.

The best known of these are Ehrlich's and Delafield's hæmatoxylin, while Mayer's hæmalam is also exceedingly useful.

(a) *Ehrlich's Hæmatoxylin.*

| | |
|---|---|
| i. Hæmatoxylin ... ... ... | 2 grammes. |
| Absolute alcohol ... ... ... | 60 c.c. |
| ii. Glycerin ... ... ... ... | 60 c.c. |
| Aq. destill. ... ... ... | 60 c.c. |
| Glacial acetic acid ... ... | 3 c.c. |

Saturate with ammonium alum.

Mix i. and ii. Allow to ripen, filter, and use.

(b) *Delafield's Hæmatoxylin.*

| | |
|---|---|
| Hæmatoxylin crystals ... ... | 4 grammes. |
| Alcohol, 95 per cent. ... ... ... | 25 c.c. |

When dissolved add 400 c.c. of a saturated aqueous sol. of ammon. alum. Expose to light and air for several days; filter, and add the following:

| | |
|---|---|
| Glycerin ... ... ... ... | 100 c.c. |
| Alcohol, 95 per cent. ... ... ... | 100 c.c. |

Filter; keep in glass-stoppered bottle.

(c) *Mayer's Hæmalam.*

| | |
|---|---|
| i. Hæmatin ... ... ... ... | 1 gram. |
| Alcohol, 90 per cent. ... ... | 50 c.c. |
| Alum ... ... ... ... | 50 grammes. |
| ii. Aq. destill. ... ... ... ... | 100 c.c. |

Mix i. and ii. After cooling, filter.

Eosin Solutions.

(a) *Alcoholic.*

| | |
|---|---|
| Eosin ... ... ... ... ... | 5 grammes. |
| Aq. dest. ... ... ... ... | 30 c.c. |
| Abs. alcohol ... ... ... ... | 70 c.c. |

(b) *Aqueous.*

| | |
|---|---|
| Eosin ... ... ... ... | 0.5 to 1 gram. |
| Aq. dest. ... ... | 100 c.c. = ½ or 1 per cent. |

To stain. Hæmatoxylin or hæmalam 5 to 20 minutes (depending on ripeness of solution); wash in water, leave there for one minute or longer to bring out the blue colour. Dry and mount, or, if preferred, counter-stain with either of the above eosin solutions for half a minute.

(2) Staining with Leishman's modification of Romanowsky's stain. As this is the most practicable of the many modifications of this stain, it is the only one that need be described here. Films are prepared in the usual manner; they are not fixed as in (1), the stain being poured on direct. (The methyl alcohol in which the stain is dissolved does the fixing, the two processes of staining and fixing going on together.)

To Stain.

1. Leave stain on for one minute.
2. Add an equal quantity of distilled water, and leave this on for 5 minutes.
3. Wash with distilled water, and leave a drop of this on for one minute.
4. Dry by blotting or in the air; mount.

